# Alpha-1-Acid Glycoprotein Quantification via Spatial Proximity Analyte Reagent Capture Luminescence Assay: Application as Diagnostic and Prognostic Marker in Serum and Effusions of Cats with Feline Infectious Peritonitis Undergoing GS-441524 Therapy

**DOI:** 10.3390/v16050791

**Published:** 2024-05-16

**Authors:** A. Katrin Helfer-Hungerbuehler, Andrea M. Spiri, Theres Meili, Barbara Riond, Daniela Krentz, Katharina Zwicklbauer, Katharina Buchta, Anna-Maria Zuzzi-Krebitz, Katrin Hartmann, Regina Hofmann-Lehmann, Marina L. Meli

**Affiliations:** 1 Clinical Laboratory, Department of Clinical Diagnostics and Services, Center for Clinical Studies, Vetsuisse Faculty, University of Zurich, CH-8057 Zurich, Switzerland; aspiri@vetclinics.uzh.ch (A.M.S.); tmeili@vetclinics.uzh.ch (T.M.); briond@vetclinics.uzh.ch (B.R.); regina.hofmann-lehmann@uzh.ch (R.H.-L.); mmeli@vetclinics.uzh.ch (M.L.M.); 2 LMU Small Animal Clinic, Centre for Clinical Veterinary Medicine, LMU Munich, D-80539 Munich, Germany; d.krentz@medizinische-kleintierklinik.de (D.K.); k.zwicklbauer@medizinische-kleintierklinik.de (K.Z.); buchta.kati@gmail.com (K.B.); a.zuzzi-krebitz@medizinische-kleintierklinik.de (A.-M.Z.-K.); hartmann@lmu.de (K.H.)

**Keywords:** alpha-1-acid glycoprotein, AGP, biomarker, acute-phase proteins, FIP, FCoV, GS-441524, cat, SPARCL^TM^ assay

## Abstract

Until recently, the diagnosis of feline infectious peritonitis (FIP) in cats usually led to euthanasia, but recent research has revealed that antiviral drugs, including the nucleoside analog GS-441524, have the potential to effectively cure FIP. Alpha-1-acid glycoprotein (AGP) has been suggested as a diagnostic marker for FIP. However, AGP quantification methods are not easily accessible. This study aimed to establish a Spatial Proximity Analyte Reagent Capture Luminescence (SPARCL^TM^) assay on the VetBio-1 analyzer to determine the AGP concentrations in feline serum and effusion samples. Linearity was found in serial dilutions between 1:2000 and 1:32,000; the intra-run and inter-run precision was <5% and <15%, respectively; and AGP was stable in serum stored for at least 8 days at room temperature, at 4 °C and at −20 °C. Cats with confirmed FIP had significantly higher serum AGP concentrations (median: 2954 µg/mL (range: 200–5861 µg/mL)) than those with other inflammatory diseases (median: 1734 µg/mL (305–3449 µg/mL)) and clinically healthy cats (median 235 µg/mL (range: 78–616 µg/mL); *p*_KW_ < 0.0001). The AGP concentrations were significantly higher in the effusions from cats with FIP than in those from diseased cats without FIP (*p*_MWU_ < 0.0001). The AGP concentrations in the serum of cats with FIP undergoing GS-441524 treatment showed a significant drop within the first seven days of treatment and reached normal levels after ~14 days. In conclusion, the VetBio-1 SPARCL^TM^ assay offers a precise, fast and cost-effective method to measure the AGP concentrations in serum and effusion samples of feline patients. The monitoring of the AGP concentration throughout FIP treatment provides a valuable marker to evaluate the treatment’s effectiveness and identify potential relapses at an early stage.

## 1. Introduction

Feline coronaviruses (FCoVs) are enveloped RNA viruses that belong to the family of Coronaviridae in the genus Alphacoronavirus. FCoVs are endemic in domestic cats. The prevalence of FCoV infection in cats is up to 90%, with the highest rates in multi-cat environments [1]. The most common form of FCoV infection results in no or only mild gastrointestinal signs [2]. Cats infected with FCoV usually shed the virus in their feces, which can then be transmitted to other cats through the fecal–oral route [3]. The majority of cats infected with FCoV experience no significant health problems, and the infection resolves on its own without a specific treatment. However, in up to 10% of cases, feline infectious peritonitis (FIP) develops [4,5]. FIP is a severe and, if untreated, often fatal form of a disease caused by FCoV infection. It occurs when highly virulent FCoVs arise de novo via mutation from less virulent FCoVs within an individuum [6]. These mutations broaden the target cell spectrum, resulting in systemic infection, and the mutated strains are able to replicate in monocytes/macrophages effectively and sustainably [7,8,9,10]. The activation of monocytes and macrophages induced by FCoV, along with the subsequent immune-mediated processes, results in various clinical signs, such as weight loss, fever, fluid accumulation in the body cavities and neurological and ophthalmologic abnormalities. Until recently, a diagnosis of FIP almost always resulted in euthanasia, with a median survival time of only eight to nine days after diagnosis [11,12]. Various antiviral and immunomodulating FIP treatment regimens were suggested, which were not found to be effective (for a comprehensive review, see [13]). However, recently, an unlicensed nucleoside analogue, GS-441524, has been found to be promising in treating and finally curing cats with FIP [14,15]. In an FIP treatment study by the authors of this work, all eighteen cats diagnosed with FIP were successfully cured after 84 days of treatment with an oral multi-component drug containing GS-441524 (Xraphconn^®^, Mutian Life Sciences Limited, Nantong, China). Notably, none of the cats experienced any severe adverse effects [16]. Furthermore, during the extensive follow-up period of nearly one year (336 days), no relapse was observed [17].

The clinical diagnosis of FIP is still very challenging as no parameter is pathognomonic; therefore, a combination of physical examination, medical history review, and laboratory tests, such as specific and quantitative FCoV real-time RT polymerase chain reaction (RT-qPCR), is often employed to diagnose FIP. FIP diagnosis includes the ruling out of other possible causes of disease through a comprehensive clinical and laboratory evaluation.

In recent years, biomarkers have provided additional information for FIP diagnosis. In 1997, Duthie and colleagues first described the potential value of raised alpha-1-acid glycoprotein (AGP) and haptoglobin (Hp) in the diagnosis of FIP [18]. Generally, acute-phase proteins (APPs) are classified depending on the magnitude of their response to a trigger. While, in cats, AGP and serum amyloid A (SAA) have been classified as major APPs with an up to 10–100-fold increase, Hp is considered only a moderate APP (with a 2–10-fold increase). Although the AGP concentration rises rapidly in response to inflammatory processes, SAA usually shows an earlier response in cats. It should also be noted that the peak time and magnitude of an APP depend on the type of stimulus as well as the species [19].

Various reports have described the possible use of AGP in supporting FIP diagnosis, despite not being pathognomonic for FIP [20,21,22,23,24]. According to recent studies, AGP has higher diagnostic efficiency for FIP diagnosis than other APPs, such as SAA and Hp [20]. Time and reliability play an important role in FIP diagnosis as cats potentially suffering from FIP are usually very ill and there is a strong possibility of death within a few days if left untreated. With the advent of antiviral compounds capable of curing FIP, the measurement of AGP has evolved beyond being solely a diagnostic tool. It is being suggested as a parameter for the monitoring of therapeutic success [25].

The methods most commonly applied for AGP measurement include enzyme-linked immunosorbent assays (ELISAs) and radial immunodiffusion. The latter is based on a sample that diffuses radially from a well into an agar gel plate that contains a specific antiserum to feline AGP. Precipitation between the feline AGP and this antiserum results in a visible ring, which is proportional to the concentration of AGP. In addition to its diminished accessibility, as it is no longer available in Switzerland and several other countries, radial immunodiffusion involves an extended incubation period of 24 to 48 h. This delay could pose challenges when swift treatment decisions are required for severely diseased cats [24]. Alternatively, commercial ELISAs are available for the detection of AGP. However, these ELISA kits frequently involve labor-intensive procedures with multiple long incubation steps. In addition, they are typically designed for use in a 96-well format, which can be a disadvantage when quick analyses of one or a few samples of individual cats are required, as is often experienced in clinical settings.

SPARCL^TM^ (Spatial Proximity Analyte Reagent Capture Luminescence) immunoassays can be used to measure test parameters more rapidly and easily than with other immunological assays. The VetBio-1 analyzer is designed for single-sample analysis, which is an advantage, especially in point-of-care (POC) diagnostics. Typically, cats with suspected FIP are presented as single clinical cases and the diagnosis needs to be confirmed swiftly. Waiting for batch analysis is not a viable choice in such situations. SPARCL^TM^ immunoassays are based on a proximity-dependent, homogenous, chemiluminescent detection method [26], using two antibody conjugates: one to acridan, a chemiluminescent substrate, and the other to horseradish peroxidase (HRP). Through the binding of the target biomarker, the acridan and HRP conjugates are brought into proximity. The addition of hydrogen peroxide causes the HRP to catalyze the oxidation of proximal acridan. This produces a flash of luminescence, which is proportional to the target biomarker’s concentration. In the absence of the target biomarker, the acridan conjugate remains unbound and thus does not produce luminescence.

One study investigated AGP in cats with confirmed or suspected FIP during treatment [25]. This retrospective observation study evaluated medical records between 2004 and 2021. Of the 42 cats included in the study, 26 fully recovered from FIP with treatment (at least 13 of them received GS-441524 preparations), and the AGP concentrations in all of these cats decreased to normal values (≤500 µg/mL). The study stated that AGP was the most rapid and consistent marker for the identification of a full recovery and to distinguish from non-recovering cats when compared to other parameters, such as FCoV antibody titers, hyperglobulinemia, lymphopenia or anemia [25]. Addie and coworkers postulated that AGP measurement could be used to document the response to treatment and as an indicator of whether to stop FIP treatment in individual cats. Moreover, the authors suggested that the confirmation of recovery from FIP necessitated two consecutive AGP measurements (≤500 µg/mL) at least one week apart [25]. The study had some limitations. For example, the treatment of the cats was inconsistent; the choice of treatment was at the discretion of the primary veterinarian and involved various medications, such as prednisolone, meloxicam, recombinant feline interferon omega and Mutian X. Moreover, the AGP concentrations were measured using two different methods, an ELISA and radial immunodiffusion. Thus, so far, no prospective study has investigated the serum AGP concentrations in cats with confirmed FIP, receiving standardized treatment, using a consistent methodology. A very recent study by Romanelli and colleagues [27] validated a commercially available AGP ELISA for FIP diagnostics. The AGP measurement was shown to be precise and accurate and able to discriminate FIP from other diseases both in serum and effusion.

The goals of the present study were (1) to establish and evaluate the SPARCL^TM^ method for the quantification of AGP in feline serum and effusion samples; (2) to investigate the utility/suitability of AGP, measured using the SPARCL^TM^ method, as a biomarker for the diagnosis of FIP (this involves the comparison of cats with confirmed FIP, those with an unrelated inflammatory disease and clinically healthy cats); (3) to determine the AGP concentrations in a prospective study involving 18 cats with confirmed FIP undergoing regularly controlled GS-441524 treatment and compare the AGP results to the SAA levels, in order to gain insights into their respective diagnostic/prognostic implications.

## 2. Materials and Methods

### 2.1. Blood Samples

Blood samples were collected from client-owned cats with confirmed FIP (n = 54), from clinically healthy cats (n = 41) and from cats with other diseases in which FIP was excluded (n = 39). The blood was collected in EDTA tubes for FCoV RT-qPCR, in heparin tubes for plasma preparation or in tubes without an anticoagulant for serum preparation.

The 54 cats with confirmed FIP were included in two FIP treatment studies: 18 cats from a previously published study [16,17] and 36 cats from a second FIP treatment study (publication underway). Both studies were performed at the LMU Small Animal Clinic, Centre for Clinical Veterinary Medicine, LMU Munich, in Germany. They were approved by the Government of Upper Bavaria (reference number 55.2–2532.Vet_02–20–52) and by the ethical committee (reference number 261–19–03–2021 and 288–11–10–2021) of the Centre for Clinical Veterinary Medicine of LMU Munich. In addition, the owners of the cats gave written informed consent to participate in the respective study. The inclusion criteria for both studies, including the diagnosis of FIP, were described in detail by Krentz and colleagues [16]. The samples tested in the current study were collected at the time of FIP diagnosis. Of the 54 cats diagnosed with FIP, 52 had effusions. Two cats had no effusion but had neurological/ophthalmological signs, and the diagnosis was confirmed by immunohistochemistry.

The group of clinically healthy control cats (defined based on the medical history, clinical picture, and blood and serum profiles, n = 41) were presented at the LMU Small Animal Clinic, Centre for Clinical Veterinary Medicine, LMU Munich, in Germany and at the University Animal Hospital in Zurich, Switzerland. These cats were found to be clinically healthy. Their use in the study was approved by the ethical committee (reference number 261–19–03–2021) of the Centre for Clinical Veterinary Medicine of LMU Munich in Germany and by the veterinary office of the Swiss Canton of Zurich (ZH 057/2019, ZH 117/2020 and ZH 093/2023). All control cats tested negative for feline immunodeficiency virus (FIV) and feline leukemia virus (FeLV).

Samples from 39 cats with diseases other than FIP were collected for routine diagnostic purposes during their clinical presentation for unrelated reasons at the University Animal Hospital in Zurich, Switzerland between June and July 2023. The samples were selected based on their high SAA concentrations (>20 mg/L) and the presence of an inflammatory leukogram (banded neutrophils > 0.12 × 10^3^/µL); both laboratory parameters indicate the presence of an inflammatory process. The clinical and laboratory findings in these cats strongly suggested diseases other than FIP. Details of the diagnoses and ages of the cats can be found in Appendix A, Table A1. Only leftover material was used for this study from these cats; no additional volumes or samples were collected.

### 2.2. Effusion Samples

A total of 74 effusion samples were available from client-owned cats submitted for routine diagnostic purposes during clinical presentation at the University Animal Hospital in Zurich, Switzerland between March 2020 and September 2022. The samples consisted of 45 abdominal and 29 thoracal effusions. For this study, only leftover material initially collected for diagnostic purposes was used; no additional volumes or samples were collected. The samples were part of the Vetsuisse Biobank; they were collected in EDTA- or plain tubes and were stored at −80 °C until analysis.

Of the 74 effusion samples from the Vetsuisse Biobank, 37 were positive and 37 were negative in the FCoV RT-qPCR (Appendix A, Table A3 and Table A4, [28]). FCoV-negative RT-qPCR results make an FIP diagnosis improbable, suggesting another underlying disease (e.g., a neoplasm or septic effusion). The diagnoses were verified and, in these cases, the effusions with FCoV-positive results originated from cats that most likely had FIP (Appendix A, Table A3).

In addition, the AGP concentrations were also measured in twelve effusion samples on the day of diagnosis (day 0) in the FIP treatment study conducted by Krentz et al. [16].

### 2.3. Determination of FCoV Viral RNA Load in Blood and Effusion Samples

Viral total nucleic acids (TNA) were extracted from the blood and effusion samples from cats in the treatment studies using the MagNA Pure 96 and MagNA Pure 96 DNA and Viral NA SV Kit (Roche Diagnostics AG, Rotkreuz, Switzerland). TNA from routine diagnostic samples was extracted using the MagNA Pure LC2 and MagNA Pure LC Total Nucleic Acid Kit (Roche Diagnostics). The input volume was 100 µL for EDTA-anticoagulated blood and 200 µL for effusions, and the samples were eluted in 100 µL elution buffer. For each batch of extractions, negative controls were run in parallel to check for cross-contamination. Feline coronavirus RNA loads were determined using an RT-qPCR assay that detected the FCoV 7b gene, as previously described [28], with some modifications [16,29]. All samples were tested for the absence of inhibition. An FCoV RNA standard curve was run in parallel to the samples to determine the viral RNA copy number.

### 2.4. Evaluation of the SPARCL^TM^ AGP Immunoassay

The AGP concentrations were measured in the feline serum, heparin plasma and thoracic and abdominal effusion samples using the cat AGP VetBio-1 SPARCL^TM^ assay and a VetBio-1 luminometer (Veterinary Biomarkers, Inc., West Chester, PA, USA).

SPARCL^TM^ immunoassays are based on a proximity-dependent, homogenous, chemiluminescent detection method [26]. Dilution and measurement were performed with kit reagents according to the manufacturer’s recommendations (Veterinary Biomarkers). In brief, 1:2000 diluted serum, heparin plasma or effusion samples were pipetted into 12 × 75 mm borosilicate tubes (Faust Laborbedarf AG, Schaffhausen, Switzerland). The acridan/HRP conjugate (0.5 mL) was added, the samples were mixed and incubated at 22.5 °C for 45 min in the dark, before being measured in the luminometer. To avoid the chemiluminescence of the borosilicate tubes, all steps involving the borosilicate tubes were performed with reduced light (lowered window blinds and no direct artificial lights). To determine the absolute AGP concentrations, a standard was run with each batch of samples in duplicate. The read-out of the assay was given as the concentration of AGP (µg/mL) and was determined from the ratio of the blank subtracted sample’s luminescence to that of the standard (1000 ng/mL). Based on the manufacturer’s recommendations, signals stronger than a 1.5 × AGP standard signal were considered potentially saturated; thus, these samples were measured again at a dilution of 1:5000.

In the present study, we refrained from performing a comparison study as suggested by ASVCP guidelines [30], because no reference method/gold standard was available. Instead, we established reference intervals (RI) from 41 clinically healthy cats and determined clinical decision limits (cut-off values) for FIP cats. The availability of RIs and cut-off values is crucial for the assay’s application in diagnostics. Systematic error has been evaluated by performing a linearity study and random errors were assessed by testing reproducibility and repeatability. The stability study gives important preanalytical information about shipment of diagnostic samples. Importantly, the SPARCL^TM^ method uses two monoclonal feline antibodies. Therefore, cross-reactivity issues could be excluded.

The linear range was determined by measuring a pooled sample consisting of various serum samples from cats diagnosed with FIP in triplicate. Two-fold serial dilutions were performed (undiluted to 1:16), which was followed by the typically performed 1:2000 dilution for each of the dilutions.

The intra-run precision was assessed in a single assay in two serum samples: one with a low AGP concentration with an average of 208 µg/mL (healthy control) and the other with an elevated AGP concentration with an average of 2920 µg/mL (cat with FIP). The AGP concentrations were determined in both samples in eight measurements.

The inter-run precision was evaluated using a serum sample from a healthy cat with low AGP and a pooled FIP sample (see above) stored at room temperature (22.5 °C) on five subsequent days and day eight. To study the stability of AGP, the samples were stored at 22.5 °C, at 4 °C, and at −20 °C in triplicates for up to 8 days between measurements.

For the AGP measurements in serum and heparin plasma, a reference value was calculated based on the AGP measurements from the serum of 41 healthy cats. Furthermore, 3000 µg/mL [22] and additional cut-offs determined in this study were tested. For the effusion samples, the best cut-off value according to the ROC analysis was determined.

### 2.5. Time Course of AGP Concentrations in Serum

Samples were available from 18 cats diagnosed with FIP and undergoing treatment at the LMU Small Animal Clinic, Centre for Clinical Veterinary Medicine, LMU Munich, in Germany (see also Section 2.1). The cats had been treated orally with Xraphconn^®^ (a multi-component drug containing GS-441524 [16]) for 84 days (day 0 to day 83) [16] and were followed up for an extended period of up to 336 days [17]. Cats with ocular or neural signs received 10 mg/kg, while the other cats received 5 mg/kg [16]. The AGP concentrations were quantified in serum samples collected from the cats on days 0 (FIP diagnosis, baseline), 2, 4, 7, 14, 28, 56 and 83 and during the follow-up period on days 168, 252 and 336. The samples were stored at −80 °C before analysis.

### 2.6. Feline Serum Amyloid A (SAA)

SAA was determined in the 18 cats diagnosed with FIP and undergoing treatment (see also Section 2.1) using a latex agglutination turbidimetric immunoassay reaction (LZ Test SAA, Eiken Chemical Co., Ltd., Tokyo, Japan) on a Cobas^®^ c 501 clinical chemistry analyzer (Roche Diagnostics AG, Rotkreuz, Switzerland). The RI was 0–3.9 mg/L [31]. Owing to a change made to the calibrator by the company in August 2022, a direct comparison between samples measured before this date and those measured thereafter was not possible. Comparative measurements were performed with both calibrators. It was shown that the deviation within the low range was small. Thus, the RI was not changed. However, there were differences in the higher measurement range, so that no direct comparisons were possible. As the change in calibrators took place during the current study, early measurements were designated as SAA and, subsequent measurements performed after the calibrator change in August 2022, SAA2.

### 2.7. Statistics

Statistical analyses were performed using GraphPad Prism for Windows, Version 10.1.2 (GraphPad software, San Diego, CA, USA). Differences between any two groups were tested for significance using the non-parametric Mann–Whitney U-test for unpaired samples (*p*_MWU_). Differences among three groups were analyzed using the non-parametric Kruskal–Wallis one-way ANOVA by ranks (*p*_KW_) for unpaired samples, followed by Dunn’s multiple comparison test (*p*_D_). The time course of the AGP concentrations within a group was analyzed using a mixed-effects model (REML) for paired samples without assuming sphericity (Greenhouse-Geisser correction), followed by the Bonferroni’s multiple comparison test. A *p*-value less than 0.05 was considered significant. For AGP, the receiver operating characteristic (ROC) curve and the area under the curve (AUC) were calculated. RI was calculated using Analyse-it on Microsoft Excel version 2018 (Build 14326.20404).

## 3. Results

### 3.1. Establishment and Evaluation of AGP Measurement Using the VetBio-1 SPARCL^TM^ Assay

During the establishment of the AGP measurements using the SPARCL^TM^ method and the VetBio-1 for the read-out, the blank values (control measurements without clinical material, providing a baseline) yielded repeatedly variable results. They ranged from <2000 relative light units (RLU), considered a normal background level, to 48,000 RLUs. After considering different factors, it became evident that, in particular, measurements performed on benches next to the window on sunny days produced increased RLU levels, even for blank values. Moreover, we observed that borosilicate tubes from different manufacturers yielded different results. By reducing the ambient and artificial light during sample preparation, including all steps involving the borosilicate tubes, and using tubes from only one source, this problem was circumvented, and reliable measurements could be achieved under standardized conditions.

**Figure A1:**
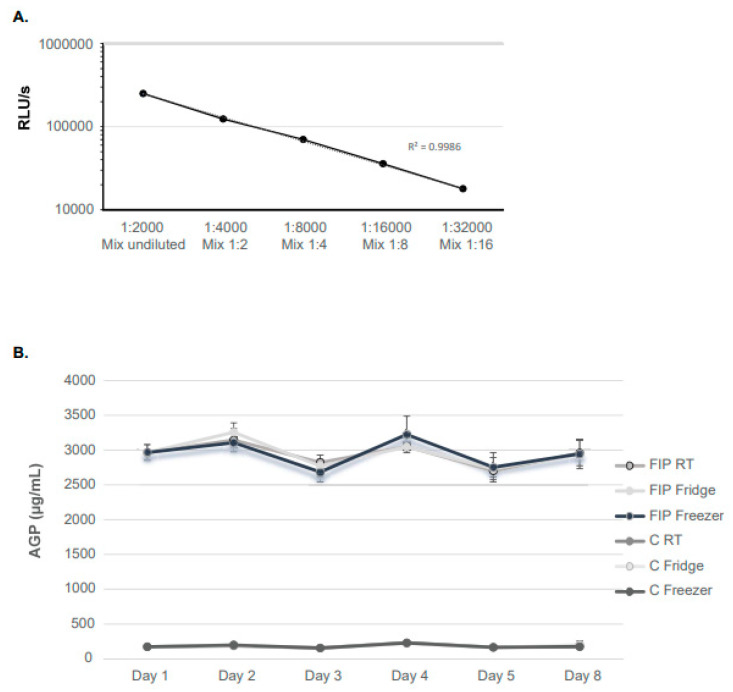
Validation of the feline α1-acid glycoprotein (AGP) assay measured on the VetBio-1. (**A**) Linearity is depicted for a sample consisting of pooled serum from cats with confirmed FIP. Serum dilution between 1:2000 and 1:32,000 revealed reproducible results, with an R^2^ = 0.9986. The coefficient of variation over all dilutions from the average of the triplicate dilutions was 5.9%. (**B**) AGP’s stability was assessed at 22.5 °C (RT), 4 °C (fridge) and −20 °C (freezer) for 8 days in an FIP and control serum sample. RLU/s (relative light units per second), C (control serum sample of a clinically healthy cat), FIP (pooled serum sample from cats with FIP).

When implemented in serum samples, the AGP SPARCL^TM^ assay’s result was linear over the entire range of dilutions measured: 1:2000–1:32,000 (representing AGP concentrations from 1.3 µg/mL down to 85 ng/mL; Appendix A, Figure A1A with an R^2^ = 0.9986). The coefficient of variation (CV) for the intra-run precision was 2.2% for the sample with a low AGP concentration (reference serum) and 4.8% for the sample with a high AGP concentration (sample from a cat with FIP). The CVs for the inter-run precision were 13.9% in the control sample and 4.8% in the FIP sample pool. AGP concentrations were shown to be stable for 8 days at all three tested storage temperatures (at 22.5 °C, 4 °C and −20 °C) (Appendix A, Figure A1B and Supplementary Table S1).

AGP concentrations in the serum of 41 healthy cats were used to establish the RI. The right-sided RI was 567 µg/mL (90% CI 411–616).

### 3.2. Comparison of AGP Concentrations in Serum of Cats with FIP, Diseased Cats without FIP and Healthy Cats

**Figure 1:**
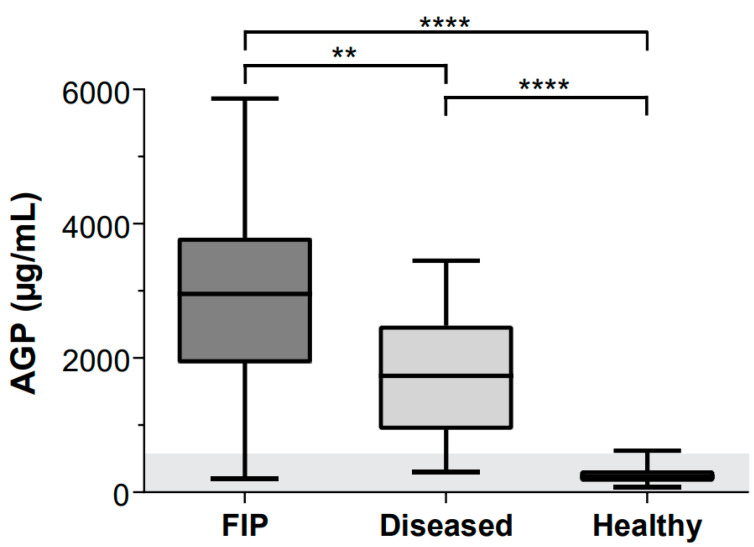
Comparison of serum α1-acid glycoprotein (AGP) concentrations in cats with FIP (n = 54), diseased cats without FIP with elevated SAA levels and inflammatory leucogram (n = 39) and healthy cats (n = 41). Serum AGP concentrations were tested for significant differences between three groups via Kruskal–Wallis one-way ANOVA by ranks (*p*_KW_ < 0.0001) and subsequently via Dunn’s post hoc test: ** = *p* = 0.0022; **** = *p* < 0.0001. Data are shown as box plots, and boxes extend from 25th to 75th percentile. Horizontal line represents median, and whiskers extend from smallest to largest value. AGP reference interval (RI) is depicted in light gray (<567 µg/mL).

The serum AGP concentrations in cats with FIP were significantly higher than those in diseased cats without FIP and clinically healthy cats (*p*_KW_ < 0.0001, Figure 1, Table 1). The median AGP concentrations were 235 µg/mL in the clinically healthy cats, 1734 µg/mL in the diseased cats without FIP and 2954 µg/mL in the cats with FIP. The serum AGP concentrations of five cats with septic effusions were significantly higher (median 2733 µg/mL) than those of the remaining diseased cats without FIP (1452 µg/mL; *p*_MWU_ = 0.0346) and nearly as high as the median concentration in cats with FIP (2954 µg/mL, Appendix A, Table A1).

**Figure 2:**
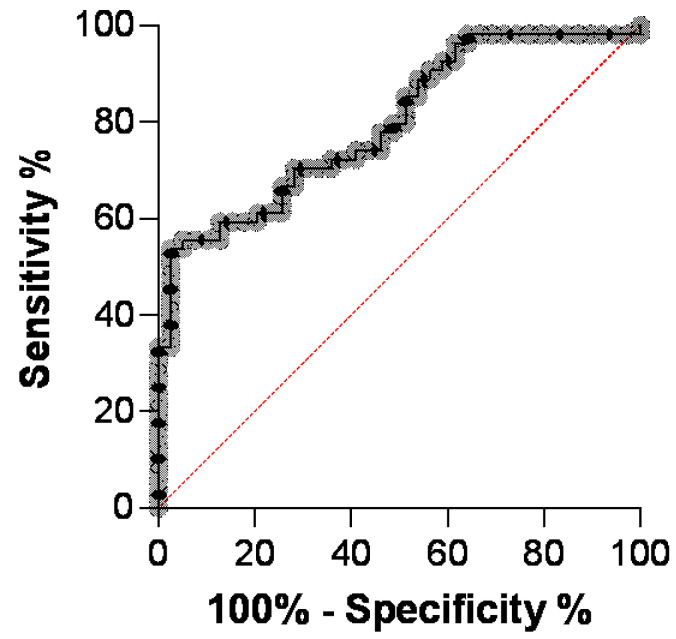
Receiver operating characteristic (ROC) curve of acute-phase protein (APP) α1-acid glycoprotein (AGP) concentrations in serum samples from cats with feline infectious peritonitis (FIP; n = 54) and diseased cats without FIP (n = 39). The gray dotted line represents AGP in serum and the thin red line shows the no-discrimination line.

A receiver operating characteristic (ROC) curve was plotted for the AGP concentration in the serum to diagnose FIP. When the ROC curve for AGP in serum was obtained using the 39 samples from diseased cats without FIP and the 54 samples from cats with FIP, an AUC of 0.80 was calculated (Figure 2; Appendix A, Table A2). A cut-off value of 2531 μg/mL showed sensitivity of 61% and specificity of 79%. Upon raising the cut-off to 2927 μg/mL, the sensitivity dropped to 54%, while the specificity increased to 97%.

### 3.3. Comparison of AGP Concentrations in Effusion Samples of Cats with FIP and Diseased Cats without FIP

**Figure 3:**
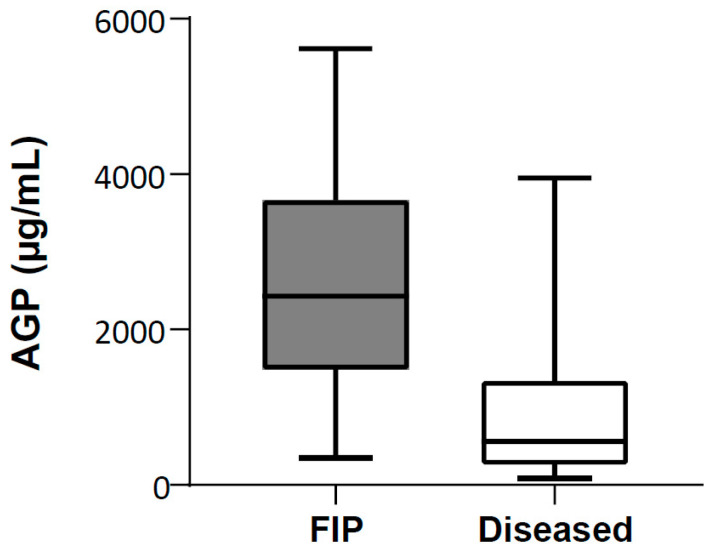
Comparison of α1-acid glycoprotein (AGP) concentrations in effusions of cats with FIP (n = 49) and effusions of diseased cats without FIP (n = 37). AGP concentrations were tested for significant differences using non-parametric Mann–Whitney U-test for unpaired samples (*p*_MWU_ < 0.0001). Data are shown as box plots, and boxes extend from 25th to 75th percentile. Horizontal line represents median, and whiskers extend from smallest to largest value.

The AGP concentrations in the effusions from cats with FIP were statistically significantly higher than the AGP concentrations in the effusions from diseased cats without FIP (*p*_MWU_ < 0.0001, Figure 3 and Table 2). The median AGP concentration in the effusions from diseased cats without FIP was 560 µg/mL, whereas that in the effusions from cats with FIP was 2425 µg/mL.

**Figure 4:**
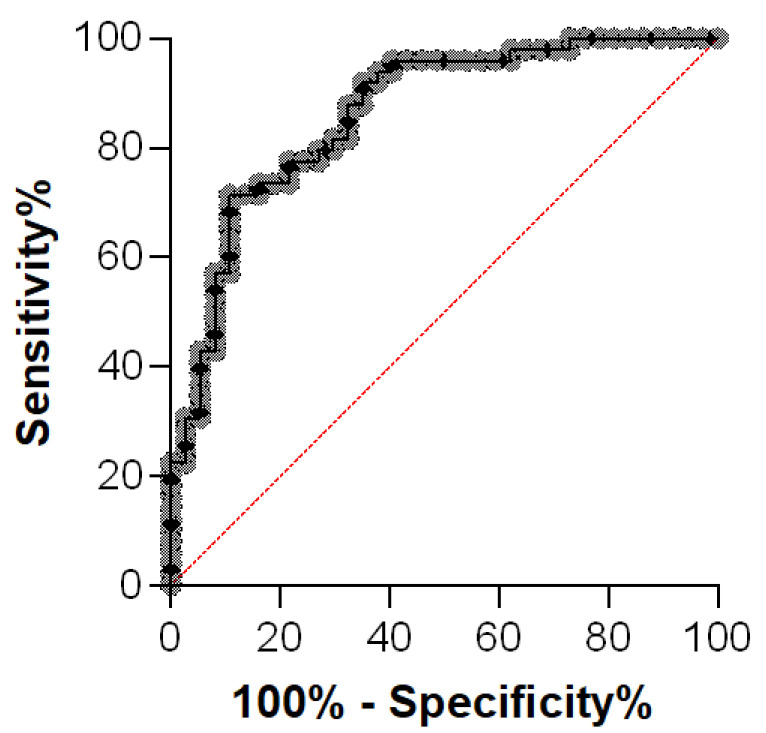
Receiver operating characteristic (ROC) curve of α1-acid glycoprotein (AGP) in effusions from cats with FIP (n = 49) and those from diseased cats without FIP (n = 37). The gray dotted line represents AGP in effusion and the thin red line shows the no-discrimination line.

An ROC curve was plotted for the AGP measurements from the 49 effusions of cats with FIP and those of 37 diseased cats without FIP. The ROC curve resulted in an AUC of 0.86 (Figure 4). A cut-off value of 1686 μg/mL for AGP resulted in the best values: sensitivity of 71% and specificity of 89%.

### 3.4. AGP Concentrations during GS-441524 Treatment of Cats with FIP

**Figure 5:**
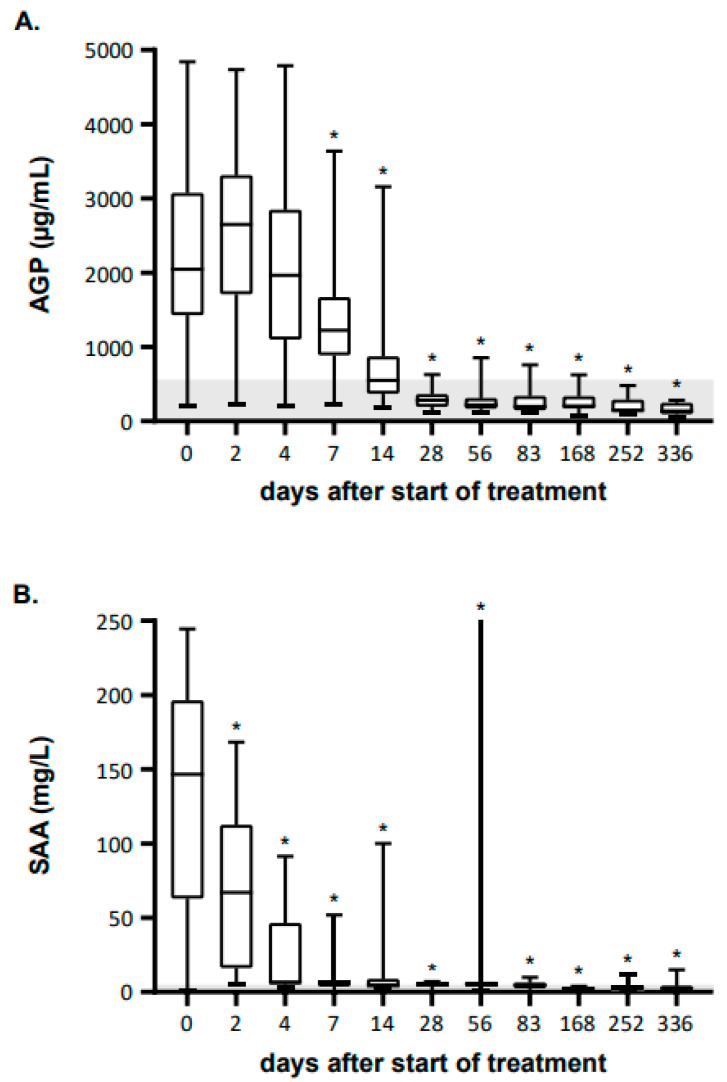
Alpha-1-acid glycoprotein (AGP) and serum amyloid A (SAA) concentrations measured during 84 days of GS-441524 treatment and at three follow-up examinations (days 168, 252 and 336 [16]). (**A**) Serum AGP concentrations. (**B**) SAA serum concentrations. Data are shown as box plots; boxes extend from 25th to 75th percentiles. Horizontal line represents median, and the whiskers extend from smallest to largest value. Asterisks mark significant differences (*p* < 0.05) of the parameters on different days of treatment when compared to day 0 (baseline before treatment) measured by a mixed-effects model. Reference values are depicted in light gray (for AGP: <567 µg/mL; for SAA: 0–3.9 mg/L).

**Figure 6:**
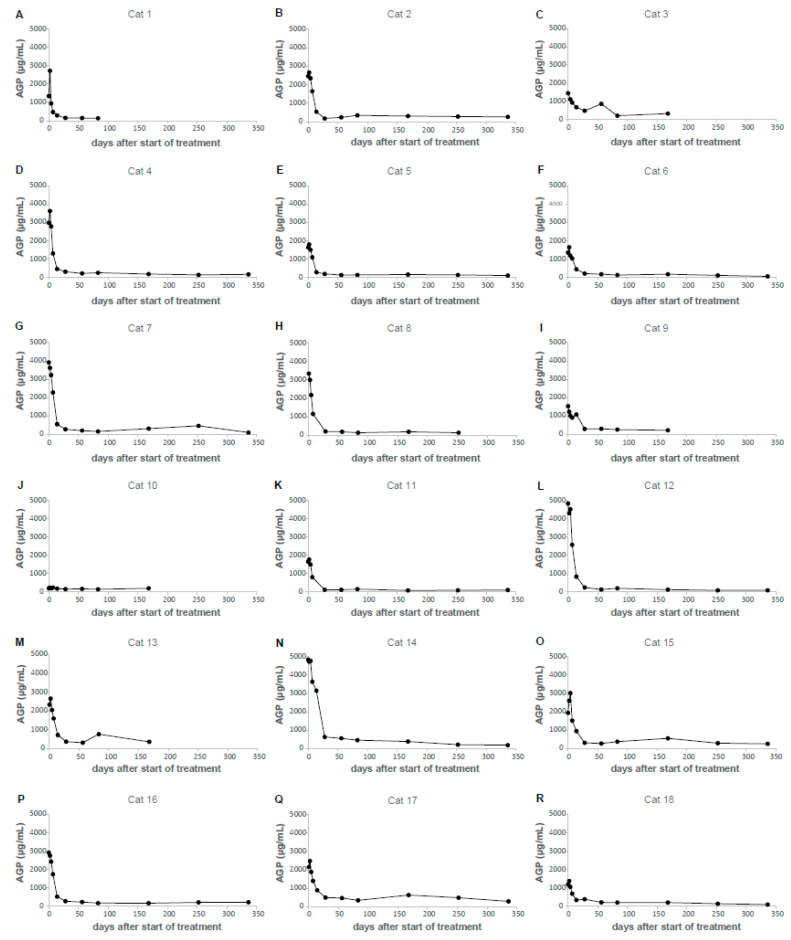
Timeline visualizing α1-acid glycoprotein (AGP) decline throughout study course in cats with FIP treated with GS-441524 [16]. (**A**) Cat 1. (**B**) Cat 2. (**C**) Cat 3. (**D**) Cat 4. (**E**) Cat 5. (**F**) Cat 6. (**G**) Cat 7. (**H**) Cat 8. (**I**) Cat 9. (**J**) Cat 10. (**K**) Cat 11. (**L**) Cat 12. (**M**) Cat 13. (**N**) Cat 14. (**O**) Cat 15. (**P**) Cat 16. (**Q**) Cat 17. (**R**) Cat 18.

The AGP concentrations were retrospectively measured in serum samples from all 18 cats participating in a treatment study [16], before, during and after treatment with GS-441524. While the AGP concentrations in these 18 cats increased from day 0 to day 2 (the median rose from 2049 µg/mL to 2651 µg/mL, respectively), on the following days of treatment, the AGP concentrations decreased continuously. A significant reduction in the AGP concentration could be found starting from day 7 and all following examination days compared to day 0 (baseline before treatment, Figure 5A). On day 28, 17/18 cats showed AGP values within the RI (<567 µg/mL). Cat 14 showed a very steep decline in AGP between day 14 and day 28, when it reached 631 µg/mL. On day 56 (the next follow-up), an AGP concentration within the RI (562 µg/mL) was observed. Three additional cats (cats 3, 13 and 17) showed a single slightly increased serum AGP concentration during the long-term follow-up period (857, 759 and 628 µg/mL). A timeline visualizing the AGP decline for each cat throughout the study course is depicted in Figure 6. With the exception of cat 10, in which the AGP concentrations were stable at very low levels, all AGP concentrations were lower on day 7 after the start of treatment compared to the beginning of the measurements.

The concentration of SAA, a major APP, decreased significantly starting from day 2 following treatment, thus faster than AGP [16]. In most cases, the SAA concentrations were within or at least very close to the RI (0–3.9 mg/L) after 4 to 7 days (Figure 5B), while most cats showed AGP concentrations within the RI (<567 µg/mL) after 28 days.

## 4. Discussion

FIP diagnosis is often challenging and relies on a variety of clinical and laboratory parameters [5]. Biomarkers such as SAA and AGP have become increasingly valuable in providing an additional layer of information. Since the remarkable discovery of effective drugs to treat cats with confirmed FIP, fast diagnosis has gained importance. In the present study, a SPARCL^TM^ assay and the VetBio-1 analyzer were investigated for their suitability to measure AGP concentrations reliably and quickly and as potential diagnostic and/or prognostic biomarkers in cats with suspected FIP and treated cats.

Feline AGP concentrations were previously determined using radial immunodiffusion [24], a method with limited availability that involves very long incubation periods, thereby preventing rapid access to the results. Commercially available ELISA kits for the detection of AGP are usually designed for and profitable only when many samples are run in parallel.

With the development of the VetBio-1, a suitable device has been introduced, which is optimal for single-sample analysis using the SPARCL^TM^ method. This holds significant promise in FIP diagnostics. The fast diagnosis of FIP in cats is crucial to be able to start treatment as soon as possible; otherwise, the clinical picture might deteriorate very quickly, and treatment may no longer be effective. This method includes sample dilution and a 45 min incubation step. Consequently, the SPARCL^TM^ assay using the VetBio-1 equipment proved to be straightforward to execute, requiring minimal hands-on time and offering a relatively short turnaround time. An additional significant advantage is the very small sample volume (4 µL) needed for the assay using the VetBio-1.

The issue of high background chemiluminescence signals, initially identified in the present study as an obstacle to the appropriate measurement of AGP concentrations and originating from the borosilicate tubes, was ultimately resolved by reducing the levels of ambient and artificial light during the samples’ preparation, incubation and measurement. The impact of chemiluminescence varied depending on the manufacturer of the tubes and therefore should be considered when establishing a standardized protocol and performing the assay.

SPARCL^TM^ assays have been used to quantify haptoglobin, C-reactive protein, AGP and Ceruloplasmin in the plasma of healthy dogs and dogs with pyometra. The assays showed good precision (intra-assay CVs ranging from 1.4% to 6.5% and inter-assay CVs ranging from 1.7% to 13.3%) [32]. In the present study, similar intra-run and inter-run precision values for AGP determination in feline serum samples using the SPARCL^TM^ assay and the VetBio-1 were found, i.e., <5% and <15%, respectively. Interestingly, the intra-run assay CV was lower for the reference sample with a low AGP concentration compared to the sample from the FIP cat with a high AGP concentration. This seems contradictory; however, it could be explained by the additional dilution step performed in the samples with high AGP concentrations. Thus, the higher CVs in samples from FIP-diagnosed cats might be a result of the additional pipetting and not the measurement itself. For the quantification of AGP, all samples need to be diluted to at least 1:2000. This high dilution of the original sample makes the interference of hemolysis, lipemia or hyperbilirubinemia highly unlikely. In addition, AGP was shown to be stable in serum at various temperatures (room temperature, 4 °C and −20 °C) for at least 8 days; this suggests its suitability for clinical applications, where samples often need to be shipped to the laboratory and are not stored at −80 °C. In addition, preliminary data show good stability (CV < 15%) for samples stored at −20 °C and repeatedly measured, as well as repeatedly defrosted over 13 months (quality control samples). A recent study [27] validating the AGP measurements in serum and effusion using an ELISA method, showed similar intra- as well as inter-assay CVs. Results in both studies were able to discriminate FIP from other diseases based on AGP concentrations as judged based on ROC analysis, indicating that AGP in serum and effusions is a useful diagnostic marker. While ELISAs are useful for batch analysis, the VetBio-1 analyzer is suitable for single sample analysis.

The serum AGP concentrations among cats with a confirmed FIP diagnosis, diseased cats without FIP and clinically healthy cats were compared. All serum samples from cats diagnosed with FIP were obtained from animals participating in an FIP treatment study with stringent inclusion criteria [16]. On the other hand, the samples from cats without FIP were sourced from patients presenting at the University Animal Hospital in Zurich, Switzerland. These cats were diagnosed with other inflammatory diseases and were therefore not considered for a concurrent FIP treatment study. Thus, based on the available information, it was assumed that these cats did not have FIP. However, the diagnosis of FIP is challenging, and it cannot be entirely ruled out that a cat diagnosed with a different condition may also have concurrent FIP.

Significant differences were found in the AGP concentrations among cats with FIP, cats with inflammatory diseases other than FIP and clinically healthy cats. These findings confirm the suitability of AGP as a diagnostic marker for FIP diagnosis. Similar to the study of Paltrinieri and colleagues [22], AGP concentrations > 2927 µg/mL strongly supported a diagnosis of FIP, even in cats with a low pretest probability (clinical signs not typical of FIP). Among all serum/plasma samples from diseased cats without FIP (n = 39, Appendix A, Table A1), only one had an AGP concentration > 2927 µg/mL. This sample originated from cat S#019, which suffered from cachexia, renal disease and anemia; presented with skin fungi; and tested positive for FeLV. The cat had been adopted and imported from the Mediterranean region two months prior. At the time of presentation, the clinical signs appeared to be unrelated to FIP and no follow-up FCoV RT-qPCR had been performed. Due to its very poor condition, the cat was euthanized on the day of presentation. Thus, in this case, FIP could not be completely excluded.

The serum AGP concentrations of cats with FIP and the control group of diseased cats without FIP but with inflammatory conditions were compared in an ROC curve analysis. The ROC analysis yielded an AUC of 0.80. A cut-off value of 2531 μg/mL showed sensitivity of 61% and specificity of 79%. The values obtained in the present study are lower than previously published ones [20,22]. One of the reasons for the lower specificity might be the choice of the control group, which included five cats with septic effusions. The latter condition has previously been shown to be associated with elevated AGP concentrations both in serum and effusions [20]. The inclusion of these five cats might have diminished the diagnostic specificity of AGP in identifying cats with FIP in the current study. Indeed, the serum AGP concentrations of the five cats with septic effusions were significantly higher than those of the remaining diseased cats without FIP and nearly as high as the median concentration in cats with FIP. Thus, when comparing the AGP concentrations of these cats to those diagnosed with septic effusions, no significant difference could be determined. In addition, the study of Paltrinieri and colleagues [22] included healthy cats as well as specific pathogen-free cats in the designated non-FIP group. This might have increased their AUC. Comparing the serum AGP concentrations in FIP-diagnosed cats with those of healthy cats in the present study also yielded a much better AUC (0.99). A rather low cut-off value of 905 μg/mL provided sensitivity of 98% with specificity of 100%. This is comparable to a study by Giordano and co-workers [21], who compared a group of FIP cats to clinically healthy cats and a group of FCoV-exposed cats without clinical signs of FIP. Moreover, in this study, significantly increased AGP levels were found in cats with FIP compared to the other groups [21]. Meanwhile, 100% sensitivity and specificity was found when using AGP as a serum marker by Giori and co-workers [23]. While the number of cases was rather low (eight FIP cases and four cases without FIP), no samples with severe inflammatory disease had been included, thus potentially leading to this outstanding sensitivity and specificity. However, in reality, the attending veterinarian faces the crucial task of determining whether a clinically ill cat is afflicted with FIP. This diagnostic challenge does not involve distinguishing a suspected cat with FIP from a clinically healthy cat, but discerning FIP from a spectrum of diseases that manifest with similar clinical signs. Notably, this encompasses cases involving cats with septic effusions. The AGP concentration is thus only one component used to distinguish FIP diagnoses from other diseases [5]. According to the data from the present study, it is not the primary marker of choice when distinguishing FIP from sepsis. Nevertheless, serum AGP concentrations > 2927 μg/mL were highly indicative of FIP (97% specificity), but nearly half of the cats with FIP were not recognized (54% sensitivity), while the cut-off value of 2531 μg/mL, suggested in this study, reduced the specificity (79%) but increased the sensitivity (61%).

While most publications suggest a cut-off above which FIP diagnosis is likely, a study from Stranieri and colleagues recommended using a “negative” AGP test (<1.5 g/L) to rule out FIP [33]. In this study, AGP concentrations were measured using a radial immunodiffusion kit in fourteen cats diagnosed with FIP: while twelve cats with FIP showed high AGP concentrations (>1.5 g/L), two of the cats with FIP had values below 1.5 g/L. Indeed, also in the present study, in one serum sample obtained from the FIP treatment study as well as in two effusions from cats diagnosed with FIP, the AGP concentrations were even below the RI (<567 μg/mL). Thus, although AGP measurement below the established cut-off can potentially exclude FIP, it cannot be used as sole criterion.

Among the cats with FIP in the present study, 52/54 cats presented with effusions [16]. Only two cats had no effusions; both had ocular signs and one also displayed neurological signs. The serum AGP concentrations of these two cats did not differ from those of the animals with effusions. However, these numbers were too small to draw conclusions regarding whether the AGP levels differ between cats with different clinical presentations of FIP, and further analysis will be necessary to investigate this issue.

In an earlier study by Hazuchova and colleagues [20], SAA, AGP and haptoglobin in both serum and effusions were evaluated as a diagnostic tool to differentiate FIP from other diseases. The AGP concentrations in the effusions showed the best results in distinguishing cats with and without FIP. In their study, the AUC of the ROC curve was 0.95 with a cut-off value of 1550 μg/mL, yielding sensitivity and specificity of 93%.

In the present study, when comparing the AGP concentrations in the effusions from cats with FIP to those from diseased cats without FIP, the analysis revealed an AUC of 0.86. With a set cut-off value of 1686 μg/mL, the test exhibited specificity of 89% and sensitivity of 71%. Thus, although the specificity was reasonably high, the sensitivity was lower. Nonetheless, the determination of the AGP concentrations in the effusions was superior concerning the diagnostic sensitivity and specificity to that in serum samples when applying a cut-off of 2531 μg/mL. Therefore, if both the serum and effusion samples of a patient are available for the measurement of the AGP concentration, effusions should be used to differentiate among cats with and without FIP. A recent study from Romanelli and colleagues [27] also demonstrated that the discriminating power is higher in effusions. They also suggest measuring AGP in effusions rather than in serum to obtain more complete diagnostic information. Cats with septic effusions might represent an exception. In the present study, an effusion sample derived from a cat with pyothorax showed an AGP concentration of 3950 µg/mL. Thus, in distinguishing cats with FIP from cats with septic effusions, serum and/or effusion AGP concentration measurements do not appear to be the ideal diagnostic test.

From twelve cats participating in the GS-441524 FIP treatment study [16], both serum and effusion samples were obtained at the day of diagnosis. In 11 samples, similarly to the findings of Romanelli and colleagues [27], effusion AGP concentrations were similar or lower than serum concentrations.

While SAA and AGP have been discussed as diagnostic FIP markers for some time, AGP is also being investigated as a prognostic marker during FIP treatment. Due to the high costs of FIP treatment, a reduction in the therapy duration based on the AGP concentration levels has been suggested (K. Hartmann, unpublished data). In addition, it has been mentioned that a reduction in the treatment duration according to the AGP levels could reduce the risk of the early development of viral resistance against GS-441524 through the inappropriate and/or excessive use of the drug. A reduction in the treatment duration should, however, be carefully considered, and suitable parameters for control should be defined. Therefore, the results of this study substantiate the notion that AGP could be an important factor contributing to this decision. In a retrospective observational study, AGP was described as a prognostic factor for FIP treatment success [25]. However, this study included samples obtained without a strict blood collection schedule and the treatment protocols were variable. Nevertheless, it was noted that, among the 16 cats exhibiting remission, defined as a reduction or decline in clinical signs, none displayed AGP concentrations within the reference range for healthy cats (<500 µg/mL [25]). In contrast, those cats that fully recovered eventually attained normal AGP levels [25]. In the present study, most of the 18 cats undergoing GS-441524 treatment [16,17] had highly elevated serum AGP concentrations during early treatment up to day 7, but the AGP concentration decreased by day 28 and remained mostly within the reference interval (<567 µg/mL). The AGP concentrations remained low, the cats remained clinically healthy, and no relapse was observed [17].

In this study, the AGP concentrations usually dropped within one week of GS-441524 treatment, supporting the treatment’s success. Normal AGP concentrations were restored within 28 days in 17 out of the 18 cats monitored during treatment. In one cat, however, namely cat 10, no change in AGP or SAA levels was observed. This cat was diagnosed with FIP based on immunohistochemistry and was unique due to its additional intestinal parasite infestation (*Giardia* spp.). The AGP and SAA concentrations remained low during the entire treatment period, as well as during the follow-up. In addition, none of the sampled time points exhibited a positive FCoV RT-qPCR result in the blood or feces (no effusions were available for qPCR).

Generally, the SAA concentrations returned to concentrations within the reference interval more quickly than the AGP concentrations. This might be due to the relatively long half-life of serum AGP, which, in humans, is five days [34], whereas the half-life of SAA in humans is only ~35 h [35]. This rather long half-life might also explain why, in some cats with FIP, the AGP concentrations were higher on day 2 of FIP treatment compared to day 0. Thus, it might be beneficial to repeat the AGP concentration measurement in unclear cases on the following day.

The possibility of a curative treatment for FIP, a previously fatal infectious disease, is also changing diagnostics. As treatment should be started as quickly as possible, it is essential that a diagnosis is made. While, previously, the diagnosis of FIP confronted pet owners with the decision to implement euthanasia, financial questions regarding the coverage of treatment costs are now receiving more attention. The sooner a treatment is started, the more likely it is to be successful and, in most cases, the faster the recovery. As a result, the search for specific and particularly sensitive biomarkers and their efficient and simple measurement is now in the foreground.

## 5. Conclusions

SPARCL^TM^ technology allows for rapid biomarker analysis with the VetBio-1 analyzer, being ideal for single-sample analysis. The method is easy to perform, is cost-effective and has been shown to be precise in measuring biomarkers such as AGP. The applied methodology proved to be suitable for the quantification of feline AGP concentrations in serum and effusion samples. AGP was shown to be a beneficial biomarker and an additional diagnostic tool to support FIP diagnosis. Moreover, AGP concentrations in serum returned to low levels with successful FIP treatment.

Further studies are necessary to determine whether AGP has potential as a prognostic marker for FIP treatment outcomes and as a helpful indicator to determine whether a shortened treatment protocol can be applied.

## Figures and Tables

**Table 1:** Descriptive statistics for measured serum α1-acid glycoprotein (AGP) in feline patients grouped by clinical disease status.

Group	N	Q1 (µg/mL)	Median (µg/mL)	Q3 (µg/mL)	IQR (µg/mL)	Range (µg/mL)
FIP	54	1997	2954	3755	1759	200–5861
Diseased without FIP	39	950	1734	2428	1478	305–3449
Healthy	41	157	235	258	100	78–616

Q: quartile, IQR: interquartile range.

**Table 2:** Descriptive statistics for measured α1-acid glycoprotein (AGP) concentrations in effusions of cats with FIP and effusions of diseased cats without FIP.

Group	N	Q1 (µg/mL)	Median (µg/mL)	Q3 (µg/mL)	IQR (µg/mL)	Range (µg/mL)
FIP	49	1554	2425	3609	2055	343–5611
Diseased without FIP	37	308	560	1329	1021	83–3950

Q: quartile, IQR: interquartile range.

## Data Availability

The authors confirm that all data analyzed in this study are available from the corresponding author upon reasonable request.

## Supplementary Materials

The following supporting information can be downloaded at: https://www.mdpi.com/article/10.3390/v16050791/s1, Table S1: Stability over time of α1-acid glycoprotein (AGP) concentrations in serum of a control sample and an FIP sample pool stored at different temperatures.

## Appendix A

**Table A1:** Information on the parameters and final diagnoses of the diseased cats without FIP.

Cat Name ^1^	Age (Years)	Sex ^2^	AGP ^3^	SAA2 ^4^	BN ^5^	Diagnosis
S#001	12.5	mn	2278	99.3	1.40	Mast cell tumor
S#002	0.7	mn	1838	105.5	1.85	Femur fracture
S#003	9.3	fn	1734	79.1	2.84	Septic effusion
S#004	0.9	mn	1128	86.2	0.27	Bite wound
S#005	13.1	mn	1225	57.2	0.94	Urinary retention problems
S#006	1.4	mn	1430	100.9	0.27	Pancreatitis
S#007	13.8	mn	595	58.2	0.37	Cardiomyopathy
S#008	0.8	fn	305	23.5	0.25	Wound infection
S#009	8.1	mn	845	70.4	0.40	Trauma (car crash)
S#010	11.5	mn	924	86.4	0.29	Cachexia, FeLV infection
S#011	1.5	mn	801	30.2	1.04	Fibular fracture
S#012	14.1	mn	2062	58.1	0.34	Abdominal wall hernia (car crash)
S#013	13.9	mn	1474	24.9	1.00	Neoplasia
S#014	7.2	fn	1959	62	0.94	Pelvic fracture, abdominal hernia
S#015	3.0	mn	2927	95.4	3.10	Head and brain trauma, bite wound
S#016	6.5	fn	1391	37.6	0.20	Intestinal foreign body
S#017	12.0	mn	2568	38.9	0.50	Gastroenteritis
S#018	2.5	mn	597	25.3	0.60	Lower urinary tract disease
S#019	5.5	mn	3449	72.6	4.17	Cachexia, renal disease, FeLV infection
S#020	7.9	mn	2668	114.5	0.21	Lymphoma
S#021	2.3	mn	590	47.3	1.04	Polytrauma
S#022	3.3	fn	967	44.9	1.08	Femur fracture
S#023	14.2	mn	2219	112	0.56	Abscess
S#024	7.0	fn	618	57.7	0.86	Radius ulna fracture
S#025	9.2	mn	2160	64.4	0.64	Traumatic abdominal hernia
S#026	14.2	fn	1143	42.1	1.08	Traumatic perianal hernia, multiple fractures
S#027	14.2	mn	2494	70.5	1.63	Multiple fractures (fall from balcony)
S#028	0.3	mn	2848	67.8	0.40	Intestinal foreign body
S#029	16.3	fn	2482	128.3	0.45	Lymphoma
S#030	6.8	fn	2375	82.4	0.96	Multiple fractures (fall from window)
S#031	12.2	mn	2733	23.4	3.59	Septic effusion, pneumothorax
S#032	1.3	fn	647	44.9	3.73	Poisoned
S#033	6.2	mn	934	63.4	1.36	Trichobezoar in the small intestine
S#034	2.3	mn	1545	20.6	0.92	Lymphadenomegaly
S#035	12.4	fn	2297	63.7	0.42	Pancreatitis
S#036	6.7	mn	2154	73.3	1.35	Septic effusion
S#037	4.0	mn	2889	67.7	6.62	Septic effusion
S#038	7.1	mn	2843	66.5	5.64	Septic effusion
S#039	12.2	fn	1160	98.2	0.71	Pyelonephritis, anemia

^1^ S: sample (serum or heparin plasma) from diseased cat without FIP, ^2^ mn: male neutered, fn: female neutered, ^3^ AGP: alpha-1-acid glycoprotein (µg/mL), ^4^ SAA: feline serum amyloid A (mg/L), ^5^ BN: banded neutrophils (×10^3^/µL; inflammatory leukogram with banded neutrophils > 0.12 × 10^3^/µL), FeLV: feline leukemia virus.

**Table A2:** Selected cut-offs showing the statistics for the receiver operating characteristic (ROC) curve plotted for serum alpha-1-acid glycoprotein (AGP) concentrations, used to diagnose FIP, using 39 diseased cats without FIP and 54 FIP-diagnosed cats.

AGP Cut-Off ^1^	Sensitivity %	95% CI ^2^	Specificity %	95% CI ^2^
448	98.2	90.2 to 99.9	2.6	0.1 to 13.2
1504	88.9	77.8 to 94.8	46.2	31.6 to 61.4
2531	61.1	47.8 to 73.0	79.5	64.5 to 89.2
2927	53.7	40.6 to 66.3	97.4	86.8 to 99.9

^1^ AGP cut-off: alpha-1-acid glycoprotein cut-off (µg/mL); ^2^ CI: confidence interval.

**Table A3:** Information on effusion samples from cats with FIP.

Cat Number ^1^	Age (Years)	Sex ^2^	FCoV RT-qPCR (Ct) ^3^	AGP ^4^	Diagnosis or Most Likely Differential Diagnosis
51052938	3.7	fn	20.3	2645	FIP
51053793	0.4	m	22.4	826	FIP
51054156	0.9	mn	22.2	717	FIP
51060583	3.4	mn	20.7	2456	FIP
51067739	0.3	m	25.8	1104	FIP
51068738	2.7	mn	17.5	5205	FIP
51069901	0.4	f	20.1	3023	FIP/pancreatitis
51070615	13.3	mn	26.6	343	FIP
51073864	2.7	mn	21.9	2866	FIP
51078145	16.8	mn	24.9	4702	FIP
51080287	3.1	mn	27.6	3570	FIP/FeLV infection
51080288	1.0	mn	25.7	3258	FIP/FeLV infection
51082265	0.9	mn	23.7	1908	FIP
51083183	9.5	mn	19.0	4768	FIP
51083366	3.9	f	21.8	2951	FIP
51083694	1.0	mn	18.3	2425	FIP
51084353	0.9	fn	26.5	1871	FIP
51085752	5.1	mn	23.6	4260	FIP
51087800	1.0	mn	20.1	4046	FIP
51088342	7.4	mn	11.8	5443	FIP
51090502	1.1	m	31.3	1976	FIP/FeLV infection
51091022	4.4	mn	29.9	2000	FIP
51093347	0.9	mn	38.1	2315	FIP
51096786	1.5	fn	22.9	1599	FIP
51098401	1.3	fn	22.5	2458	FIP
51100616	10.4	fn	19.0	4409	FIP
51101074	0.8	fn	19.7	1847	FIP/FCV
51102326	0.9	mn	24.0	971	FIP
51103629	0.4	m	18.7	5611	FIP
51111636	0.6	f	30.3	4356	FIP
51113343	15.0	fn	17.8	5374	FIP
51113346	0.9	f	19.7	3609	FIP/FeLV infection
51114606	12.7	fn	18.9	3715	FIP
51118745	1.2	mn	19.4	1424	FIP
51121069	1.9	mn	23.0	459	FIP
51121714	4.4	mn	21.8	1125	FIP
51125665	12.4	mn	22.5	1816	FIP

^1^ Unequivocally assignable sample number, ^2^ m: male, mn: male neutered, f: female, fn: female neutered, ^3^ FCoV RT-qPCR (Ct): feline coronavirus reverse-transcription quantitative polymerase chain reaction cycle threshold, ^4^ AGP: alpha-1-acid glycoprotein (µg/mL), FIP: feline infectious peritonitis, FeLV: feline leukemia virus, FCV: feline calicivirus.

**Table A4:** Information on parameters and most likely differential diagnoses of the cats with effusion samples.

Cat Number ^1^	Age (Years)	Sex ^2^	FCoV RT-qPCR (Ct) ^3^	AGP ^4^	Diagnosis or Most Likely Differential Diagnosis
51051793	13.3	m	negative	214	Suspicion of tumor
51052859	11.7	fn	negative	218	Carcinoma in the pericardium
51054322	2.0	f	negative	152	Paralytic ileus
51055437	9.8	mn	negative	450	Encephalopathy
51057024	14.6	mn	negative	1598	IBD, chronic enteropathy
51061233	1.5	mn	negative	1345	Fever and effusion of unknown etiology
51061244	11.0	mn	negative	1329	Triaditis with pot. obstructive cholestasis
51071148	12.9	mn	negative	560	Triaditis, cardiomyopathy
51072682	15.8	fn	negative	159	Neoplasia
51073972	11.0	fn	negative	1630	Chylothorax
51074362	16.6	fn	negative	1659	Hepatopathy, suspicion of tumor
51074741	0.9	f	negative	1565	Fever and effusion of unknown etiology
51083736	12.1	fn	negative	308	Chylothorax neoplastic
51085698	1.5	f	negative	114	Ovariectomy
51088349	6.0	mn	negative	459	Pulmonary thrombose
51089415	8.3	mn	negative	468	Gastrointestinal bleeding
51091461	4.9	fn	negative	2685	Renal empyema, nephrectomy
51101065	14.7	fn	negative	1226	Carcinoma
51101301	12.3	mn	negative	1147	Carcinoma
51102634	8.9	fn	negative	404	Neoplasia
51104521	0.5	m	negative	170	Cardiovascular disease
51106980	1.9	mn	negative	417	Phlebitis
51107291	11.8	fn	negative	83	Cardiomyopathy, pulmonary hypertension
51108712	0.9	fn	negative	599	Immune-mediated pancytopenia, aplastic anemia
51109370	13.5	fn	negative	116	Bronchial pneumonia
51111000	13.3	fn	negative	480	Chylothorax
51111246	11.3	mn	negative	214	Thymoma
51113179	17.4	fn	negative	837	High-grade enteropathy with secondary peritonitis
51113895	14.2	m	negative	2101	Lymphoma
51114398	0.9	mn	negative	3950	Pyothorax
51120616	8.7	mn	negative	1005	Carcinomatosis
51121032	1.9	f	negative	647	Gastroenterocolopathy
51123075	14.5	mn	negative	383	Mediastinal neoplasia
51124448	1.0	mn	negative	3336	Pancreatitis, gastroenteritis
51126811	12.4	f	negative	532	Carcinomatosis
51128014	10.3	fn	negative	564	Carcinomatosis
51128383	14.6	mn	negative	756	Neoplasia

^1^ Unequivocally assignable sample number, ^2^ m: male, mn: male neutered, f: female, fn: female neutered, ^3^ FCoV RT-qPCR (Ct): feline coronavirus reverse-transcription quantitative polymerase chain reaction cycle threshold, ^4^ AGP: alpha-1-acid glycoprotein (µg/mL), IBD: inflammatory bowel disease.
